# The role of defenders' and victims' popularity in the effectiveness of defending in bullying interactions: A longitudinal social network study

**DOI:** 10.1111/jora.70126

**Published:** 2025-12-30

**Authors:** Stefanie Richters, Maarten H. W. van Zalk, René Veenstra, Lydia Laninga‐Wijnen

**Affiliations:** ^1^ Department of Developmental Psychology University of Osnabrück Osnabrück Germany; ^2^ Department of Sociology University of Groningen Groningen The Netherlands; ^3^ INVEST/Psychology University of Turku Turku Finland

**Keywords:** bullying, defending, peer victimization, popularity, social networks

## Abstract

Peer defending is widely promoted as a strategy to reduce bullying, but few studies have investigated whether having more defenders decreases victimization over time from the victim's perspective. This social network study examined the longitudinal association between nominating more defenders and subsequent victimization among (early) adolescents and tested whether this relation is moderated by the popularity status of the defender and the victim. The sample included 1450 participants from 93 secondary school classes (grades 4–9) in Finland (52.51% female; *M*_age_ = 12.38 years, SD_age_ = 1.56). Results from longitudinal social network analyses showed that contrary to expectations, having more defenders did not reduce victimization over time. Moreover, the popularity of the defender or the victim did not moderate this effect. Results did not differ by grade. These findings suggest that defending alone may not protect students from ongoing victimization and highlight the need for broader, multi‐level intervention strategies.

Over the past decades, school bullying has gained increasing attention, and a wide range of anti‐bullying interventions have been developed. Many of these interventions focus on reducing passive bystander behavior and encouraging students to step in and support victims (Gaffney et al., 2021). However, recent findings and theoretical arguments have questioned the effectiveness of defending as a means of reducing bullying (e.g., Laninga‐Wijnen et al., 2023). Consequently, there is an emerging debate about whether defending is always beneficial. Our study uses a longitudinal social network approach to examine whether having more defenders reduces subsequent victimization from the victim's perspective and whether this effect depends on the popularity of the defender and victim at baseline.

To date, research on the effectiveness of defending in reducing bullying and victimization has yielded mixed results. Several studies have demonstrated positive effects. For example, cross‐sectional studies have shown that at the class level, there is a negative association between defending and bullying (Salmivalli et al., 2011) and between defending and victimization of vulnerable students (Kärnä et al., 2010). Additionally, observational research has shown that defending can effectively stop bullying in the moment, with bullying stopping within 10 s in over two‐thirds of cases (Hawkins et al., 2001). Longitudinal analyses at the individual level have shown that perceived increases in defending predict a decrease in self‐reported bullying over time (Saarento et al., 2015). However, a recent longitudinal study found no difference in the frequency of victimization reported by defended and non‐defended victims over time (Laninga‐Wijnen et al., 2023). In an intervention study, victims who were not assigned to a support group reported decreases in victimization over the school year, whereas those who were assigned to a support group did not (van der Ploeg et al., 2016). In addition, a large meta‐analysis of anti‐bullying interventions found that interventions including a component encouraging bystander intervention were associated with smaller mean effect sizes compared to those without such a component (Gaffney et al., 2021).

These mixed findings raise the question of whether defending is an effective means of countering bullying, which requires further investigation. This study aimed to examine the longitudinal effect of nominating more defenders on the frequency of victimization over time using a longitudinal social network approach. Despite the previous mixed findings, we hypothesized that nominating more defenders would be related to a greater decrease in victimization frequency over time (Hypothesis 1) because the rationale for encouraging defending among bystanders in anti‐bullying interventions rests on a strong theoretical foundation (Salmivalli, 2014). Bullying is often motivated by social rewards, such as visibility and popularity among peers. Bullies typically target rejected and unaccepted peers (Veenstra et al., 2010) in the presence of bystanders (Hawkins et al., 2001). If bystanders remain passive or assist the bullies, they signal their approval of the bullying, which fuels its continuation. In contrast, when bystanders defend the victim, the bullying becomes less rewarding and is more likely to stop.

The social network approach has several advantages. Rather than treating defending as an isolated individual characteristic, it treats defending as a set of directed ties within a network. A social network approach can account for interdependent relations: a victim can nominate multiple defenders, and multiple victims may share the same defenders. Moreover, this approach accounts for structural tendencies in the defending network, such as the tendency for victims with defenders to nominate additional defenders over time. It also accounts for the structural constraint that only victimized students can be defended. By accounting for the relational structure and dynamics involved in defending, it provides a complementary perspective to prior research that has used conventional regression methods.

Most importantly, the social network approach allows us to examine whether the effect of having more defenders on the frequency of victimization depends on characteristics of specific victims and of their defenders. This is important because defending may not reduce victimization for all students and may even have adverse effects for some. The second aim of this study was to test whether the effect of having more defenders on the frequency of victimization over time depends on the popularity of specific victims and their defenders at baseline. Because the network approach captures who nominates whom, it offers an ideal methodological framework for investigating how characteristics of both nominators and nominees moderate the effectiveness of defending.

We conceptualize popularity as social status within the peer group, which is associated with power, prestige, and social dominance and is typically assessed by peer nominations of who is the most and least popular in a class (Cillessen & Marks, 2011). Popularity plays an important role in the dynamics of bullying and defending (e.g., Salmivalli et al., 2021), especially during adolescence (Pouwels et al., 2018). It is important to distinguish popularity from social preference, which reflects being liked by peers. In childhood, these constructs show moderate correlations, yet they become increasingly distinct during adolescence (van den Berg et al., 2020).

Popular students are more visible than unpopular students (Cillessen & Marks, 2011) and exert greater influence among their peers (Pellegrini et al., 2011). For instance, in classes where popular students (compared to unpopular students) defended others, all students in these classes, including those who had been victimized, showed enhanced positive social–emotional adjustment outcomes (Laninga‐Wijnen et al., 2021). Bullying, by definition, involves a power asymmetry between the bully and victim (Olweus, 1993). This imbalance may be mitigated when a popular student intervenes on behalf of the victim. Due to their elevated social status, defending by popular students is likely to carry greater weight, thereby increasing the likelihood that bullies attend to and comply with their anti‐bullying message. Moreover, popular students often possess stronger social skills (Lease et al., 2002), which may enable them to respond to bullying in more socially effective and appropriate ways. Therefore, we expected that nominating popular students (compared to unpopular students) as defenders would predict a stronger decrease in the frequency of victimization over time (Hypothesis 2).

Not only the popularity of defenders but also that of victims may determine the extent to which nominating more defenders predicts a decrease in victimization. Victimization is not limited to unpopular students: popular students may also be victimized, for example, in contexts of status competition, where bullying a popular peer may be an effective way to promote one's own status (Dawes & Malamut, 2020). From the bully's perspective, there may be an increased risk associated with targeting a popular victim, as they may face social repercussions (see e.g., Salmivalli, 2010). If a popular victim is defended, this risk may be further amplified: in such cases, defending can signal strong support and admiration for the victim, reinforcing their high social standing and increasing the potential social costs of continued bullying. In contrast, unpopular victims may be perceived as safer, lower risk targets, and defending them may have less of an impact on bullying behavior. Given the lack of prior research on this topic, we explored the moderating role of the victim's baseline status in the effect of nominating more defenders on the frequency of victimization over time.

The prioritization of popularity over other relational goals peaks during adolescence (Lafontana & Cillessen, 2010). This change in social goals is due to biological (maturation and interest in romantic relationships), socio‐structural (transition to secondary school and increased importance of peer relationships), and socio‐cognitive (increased awareness about social position) changes that characterize the developmental phase of adolescence (Veenstra & Laninga‐Wijnen, 2023). Because popularity plays an increasingly important role in bullying dynamics during adolescence (Pouwels et al., 2018), we expected that in higher grades, the effect of nominating more defenders on the frequency of victimization would increasingly depend on the popularity of the defender (i.e., the moderating role of defender's popularity in the relation between nominating more defenders and victimization will be stronger in higher than in lower grades; Hypothesis 3). We also explored the effect of grade level on the interaction between the longitudinal effect of nominating more defenders on the frequency of victimization and the popularity of the victim.

## METHODS

This study's analysis code and results output are available at https://osf.io/tvrcx. Data are available upon request and data of students with consent for archiving will be publicly available after project completion (end of 2025). The study's hypotheses and analyses were preregistered at https://osf.io/sv3hk. Any divergence from the preregistration is reported in the Supporting Information S1 including a justification.

### Sample

Data were collected in Finland as part of the SOLID project, which involved two data collection cohorts, with three waves per cohort. The SOLID project design, sampling and materials were preregistered at https://osf.io/nghwm. Schools were recruited from across Finland. A 2020 national register of educational institutions was used to identify eligible schools. Secondary and non‐Finnish‐speaking schools were excluded, and all remaining schools were invited to participate. The participants were students in grades 4–9. Data were collected throughout the school year (cohort 1: 2022/2023; cohort 2: 2023/2024), with intervals of approximately 3 months (T1: September/October; T2: January; T3: April/May). Across cohorts, 713 classes participated in at least one wave of data collection. Of these, 180 classes were excluded because they did not participate in all three waves of the data collection, whereas 360 classes were excluded because less than 70% of students participated in at least one of the waves. While a participation threshold of 80% is typically recommended, a 70% cutoff can be considered acceptable (Zandberg & Huisman, 2019). Finally, 80 classes were excluded because the Jaccard index was NA for either period, which means that there were no ties to compare. Therefore, we ended up with a sample of 93 classes from 35 schools that could be included in the single‐class social network analyses (grade 4: 18 classes; grade 5: 20 classes; grade 6: 15 classes; grade 7: 20 classes; grade 8: 15 classes; grade 9: 5 classes). The sample comprised 1450 participants (52.51% female, 46.10% male, 1.38% other). The average age of participants at T1 was 12.38 years (SD = 1.56).

### Procedure

The study was approved by the Ethics Committee of the University of Turku (Finland). Prior to data collection, active consent was obtained from both parents and students. Data collection took place during regular school hours under the supervision of the teachers. Teachers received detailed instructions on the data collection during the week before each wave. The digital questionnaires took approximately 30 min to complete. Participants were informed that their responses would remain confidential and that they could withdraw their participation at any time.

### Measures

#### Frequency of victimization

Participants were provided with a definition of bullying: “It is bullying when someone is intentionally and repeatedly hurt. It is difficult for the bullied person to defend himself/herself. Bullying can be saying mean things, calling nasty names, leaving someone outside the group, hitting, pushing, telling mean things or telling lies, other acts that offend another. It is also bullying when a pupil is repeatedly teased in a mean and offensive way. Friendly and playful teasing is not bullying, nor is it when two roughly equally strong pupils argue or fight.” Then, the frequency of victimization was assessed with the global victimization item of the Olweus Bully/Victim Questionnaire (Solberg & Olweus, 2003) “How often have you been bullied by a peer at school in the last couple of months?” on a 5‐point scale (0 = “not at all”; 1 = “once or twice”; 2 = “2–3 times a month”, 3 = “once a week”, 4 = “several times a week”).

#### Defending

Participants who indicated that they had been bullied at least once or twice in the previous couple of months (i.e., scoring at least 1 on the victimization item) were asked “Whom of your classmates has defended you when you were being bullied?” They could nominate an unlimited number of students from a list of all classmates (i.e., participants and non‐participants). The participants' nominations were transformed into adjacency matrices, with victim → defender nominations coded as 1 and non‐nominations coded as 0. Non‐victimized participants could not nominate defenders and were therefore treated as structural zeros (i.e., as impossible ties, rows for victim → defender ties were coded as 10). The victim → defender ties of students who did not participate in the data collection due to missing consent were also coded as 10. Similarly, students who joined or left a class during the course of the school year were treated as structural zeros in those waves where they were not part of the class, with the corresponding rows for victim → defender ties coded as 10. Students who were absent from a specific wave of data collection for other reasons were treated as missing data, i.e., the corresponding rows were coded as NA.

#### Popularity at T1 and T2

Participants were asked “Who are the most popular?” and could nominate any number of students from a list of all classmates. The proportion of received popularity nominations was calculated for each participant, with the total number of received nominations divided by the number of potential nominators in the class (i.e., all students who answered the item). Popularity was included in the model as a varying covariate.

#### Gender

Participants indicated their gender in response to the item “I am a boy/girl/other, namely…”. Gender was included as a constant covariate in the model and was coded as 0 for boys and 1 for girls. As only a small number of participants identified as “other” (1.38%), gender was coded as NA for these participants.

### Analytic strategy

Longitudinal social network analysis was applied using stochastic actor‐based models in RSiena (Snijders et al., 2010). This modeling approach enables the investigation of the network–behavior dynamics of defending and victimization while accounting for both structural and individual‐level influences. Stochastic actor‐based models simulate the unobserved, continuous changes in network structure that occur between timepoints. These changes are conceptualized as sequences of micro‐steps, in which an actor in the network can maintain, create, or terminate a tie. The rate function determines how frequently the actors are allowed to change a tie, while the objective function determines the likelihood of specific tie changes (Snijders et al., 2010). The Method of Moments estimation was employed, as this estimation method can accommodate structural zeros in the network matrices (Ripley et al., 2024).

#### Model specification

Uniplex structural effects model the effects of the network itself on subsequent network changes. Covariate effects model the effects of actor attributes on network changes over time. These types of effects were included in the model to control for general tendencies in how defending ties are formed in the network. They were first selected based on recommendations (Ripley et al., 2024; Snijders et al., 2010) and previous findings from social network analyses of defending networks (e.g., Hooijsma et al., 2021; Huitsing et al., 2014) and then modified in a data‐driven way to ensure an adequate representation of the general tendencies of tie formation in the defending network (as recommended by Snijders et al., 2010). See the preregistration for the parameters that were initially included and the Supporting Information S2 for a description of the steps taken to reach the final solution.

First, the models were run separately for each class. The final models included the following uniplex network and covariate effects: the *outdegree effect* was estimated to capture the general tendency of actors to nominate peers as defenders. This effect reflects the density of the network (i.e., the proportion of observed ties relative to all possible ties). Although the *reciprocity effect—*representing the tendency for mutual nominations*—*is typically included by default in RSiena models, we excluded it in our study due to convergence problems in preliminary model runs. To assess the robustness of our findings, we conducted additional analyses including the reciprocity parameter, see the Supporting Information S3 (Table S3.1). The *outdegree activity effect* was included to model the tendency for actors who initially nominated many defenders to nominate even more defenders over time. Following recommendations (Snijders et al., 2010), square roots of degree values were used instead of raw counts to account for diminishing marginal effects at higher degree levels. Additionally, *the same‐gender covariate effect* was included to account for the observed tendency of actors to nominate same‐gender peers more often than other‐gender peers.

Victimization was added to the model as a behavior variable. The distributional characteristics of victimization are captured through two shape parameters that serve as control variables in the model: the *linear shape effect* functions as an intercept, indicating the average tendency toward higher or lower values, and the *quadratic shape effect* represents the self‐reinforcing dynamics of victimization (Veenstra et al., 2013).

Behavioral evolution effects model the effects of the network (here: defending network) on changes in the behavior variable (here: victimization). These effects were included in the model to test our hypotheses and exploratory analyses. In model 1, the *defending outdegree → victimization* was estimated to test our hypothesis that nominating more defenders predicts a decrease in the frequency of victimization over time (Hypothesis 1). In models 2a, 2b, and 2c, we added popularity at T1 and T2 as a varying covariate to the model. To test our hypothesis that nominating popular defenders will predict a stronger decrease in the frequency of victimization over time (Hypothesis 2), we estimated the interaction between *defending outdegree → victimization effect* and the total *defenders' baseline popularity* (model 2a). To explore whether the effect of nominating more defenders on the subsequent frequency of victimization depended on the popularity of the victim, we estimated the interaction between *defending outdegree → victimization effect* and the *victim's baseline popularity* (model 2b). Because model stability decreased and fewer classes reached convergence as more effects were added, we first estimated the model with each interaction term separately, followed by a model including both interaction terms simultaneously (model 2c).

#### Meta‐analyses across classes

The results from classes where the model converged were subsequently meta‐analyzed using the *metafor* package in R. Based on the recommendations of the RSiena manual (Ripley et al., 2024), model convergence was considered adequate if the overall maximum convergence ratio is <0.25. For each parameter, we excluded class‐level estimates that met any of the following criteria: the parameter was coded as *NA*, the standard error was equal to or greater than 10, or the parameter estimate was equal to or greater than 20 (cf. Laninga‐Wijnen et al., 2020). We added “grade level” as a multilevel continuous predictor to the meta‐analysis to test the effect of grade level on the *defending outdegree → victimization effect* moderated by the *defenders' baseline popularity* (Hypothesis 3) and the *defending outdegree → victimization effect* moderated by the *victim's baseline popularity* (exploratory analysis).

#### Goodness of fit

Goodness of fit statistics were computed for all converged classes using the *SienaGOF* function in RSiena. Three indices were assessed: the distribution of the indegree, outdegree, and triad census. These indices allow evaluation of whether the observed data fall within the distribution generated by the model simulations. Results were visualized in violin plots and statistically evaluated. Model fit is considered acceptable when the corresponding *p*‐values < .05.

## RESULTS

### Model convergence

As anticipated in the preregistration, several classes were excluded because the model did not converge. Of the 93 classes analyzed, 44 achieved model convergence for model 1. We ran logistic regressions to test the effect of class‐level predictors on model convergence (see Table 1). RSiena requires a minimum level of data density to reliably estimate social network models (Ripley et al., 2024). In our sample, the defending networks were notably sparse. Average degrees ranged from 0.00 to 2.21 in wave 1, 0.00 to 1.43 in wave 2, and 0.00 to 1.87 in wave 3. Moreover, only students who reported being victimized could nominate defenders, resulting in a high proportion of structurally impossible ties coded as structural zeros. The proportion of structural zeros among all possible ties varied substantially by class, ranging from 21% to 90% in wave 1, 33% to 92% in wave 2, and 48% to 93% in wave 3. Plots showing the probability of convergence depending on the characteristics of the defending network show that for all waves, there was a trend for classes with a higher average degree (i.e., more defending) and a lower proportion of structural zeros (i.e., more victimization) to be more likely to converge (see the Supporting Information S4). However, logistic regressions showed that only the average degree in wave 3 significantly predicted model convergence. The lack of significance in other predictors may be due to limited statistical power at the class level, given the modest sample size (*N* = 93 classes). Moreover, class size emerged as a significant predictor of model convergence, with larger classes showing a higher likelihood of convergence. Further discussion of these data exclusions is provided in the discussion section.

**TABLE 1:** Logistic regressions testing the effect of network density, proportion of structural zeros, Jaccard indices, and class size on model convergence.

	*b*	*SE*	*z*	*p*
T1 Proportion of structural zeros				
Intercept	1.739	1.075	1.618	.106
T1 proportion of structural zeros	−2.740	1.562	−1.754	.079
T1 Density				
Intercept	−0.557	0.362	−1.537	.124
T1 density	0.718	0.472	1.518	.129
T2 Proportion of structural zeros				
Intercept	1.416	1.155	1.226	.220
T2 proportion of structural zeros	−2.267	1.691	−1.340	.180
T2 Density				
Intercept	−0.418	0.390	−1.072	.284
T2 density	0.618	0.655	0.944	.345
T3 Proportion of structural zeros				
Intercept	1.639	1.374	1.193	.233
T3 proportion of structural zeros	−2.451	1.907	−1.285	.199
T3 Density				
Intercept	−0.686	0.343	−2.002	.045
T3 density	1.459	0.695	2.098	.035
T1–T2 Jaccard index				
Intercept	0.002	0.321	0.008	.993
T1–T2 Jaccard index	−0.346	0.771	−0.448	.654
T2–T3 Jaccard index				
Intercept	−0.161	0.301	−0.533	.594
T2–T3 Jaccard index	0.182	0.750	0.243	.808
Class size				
Intercept	−2.404	1.001	−2.401	.016
Class size	0.122	0.052	2.359	.018

*Note*: Logistic regressions were run separately for each variable.

Abbreviations: T1, Time 1; T2, Time 2; T3, Time 3.

Analyses for model 2 were restricted to the 44 classes in which model 1 had converged. Models converged for 28 classes for model 2a, for 33 classes for model 2b, and for 21 classes for model 2c.

### Descriptives

Table 2 displays average descriptive statistics for the defending networks. The average number of nominated defenders across all students (i.e., victims and non‐victims) was between 0.63 at T1 and 0.40 at T3. 35% of all possible ties were reciprocated at wave 1, and this proportion dropped substantively to 15% at wave 3. 39%–41% of possible triads were closed. Consistent across time points, defending nominations mostly occurred between same‐gender peers (81%–86%). The Jaccard index, which assesses network stability across two subsequent waves by capturing the proportion of stable ties relative to all ties (stable, new, and dissolved), exceeded 0.30 for either period, meeting the recommended threshold for longitudinal network analysis (Snijders et al., 2010). Hamming distances quantify the number of differing tie values between two subsequent networks and were slightly larger in the first period than in the second period.

**TABLE 2:** Descriptive statistics of defending networks per wave.

	Wave 1, *M* (SD)	Wave 2, *M* (SD)	Wave 3, *M* (SD)
Outdegree	0.63 (0.46)	0.50 (0.32)	0.40 (0.34)
SD Outdegree	1.43 (0.94)	1.22 (0.78)	1.07 (0.89)
SD Indegree	0.65 (0.28)	0.62 (0.25)	0.52 (0.26)
Reciprocity	0.35 (0.36)	0.34 (0.38)	0.15 (0.30)
Transitivity	0.41 (0.30)	0.39 (0.36)	0.39 (0.40)
Same‐gender nominations	0.86 (0.22)	0.81 (0.26)	0.81 (0.24)

	Wave 1 → 2, *M* (SD)	Wave 2 → 3, *M* (SD)
Hamming distance	7.73 (6.97)	6.31 (6.46)
Jaccard index	0.32 (0.27)	0.29 (0.28)

*Note*: *N* = 93 classes: initial sample for which social network analysis was computed.

Table 3 presents descriptive statistics for victimization, popularity, and defending across the three waves. On average, victimization levels were low (ranging from 0.51 to 0.59), with approximately one‐third of participants reporting having been victimized at least once or twice. Among those who were victimized, the majority (69%–78%) nominated at least one defender, and this proportion decreased slightly over time. Among defended victims, the average number of nominated defenders was 3.26 at T1. This count varied substantially across students and declined slightly over time. The proportion of students who were nominated as defenders at least once also declined across waves. Overall, victimization remained relatively stable across timepoints. Among those identified as victims, average victimization decreased over time. In the first period, the decrease was slightly greater among those who did not nominate a defender. In the second period, this trend reversed, with a larger reduction observed among victims who had nominated a defender. The baseline popularity levels were comparable among all students, indicating that neither the defenders nor the victims differed substantially from the classroom average.

**TABLE 3:** Descriptive statistics of victimization, popularity, and defending per wave.

	Wave 1	Wave 2	Wave 3
Number of participants who responded to victimization items	1342	1337	1308
Victimization across all participants, *M* (SD)	0.54 (0.95)	0.59 (1.02)	0.51 (1.00)
Proportion of victimized students among all participants	0.34	0.35	0.29
Proportion of victims with at least one defender	0.78	0.71	0.69
Proportion of students nominated as defender at least once	0.47	0.39	0.33
Number of defenders per defended victim, *M* (SD)	3.26 (2.73)	2.73 (2.28)	2.75 (2.65)
Number of victims defended by a defender *M* (SD)	1.42 (0.68)	1.33 (0.60)	1.25 (0.56)

	Wave 1 → 2	Wave 2 → 3
Change in victimization between waves across all participants, *M* (SD)	0.05 (0.95)	−0.07 (0.97)
Change in victimization among all victims, *M* (SD)	−0.35 (1.26)	−0.54 (1.26)
Change in victimization among victims with at least one defender, *M* (SD)	−0.32 (1.28)	−0.57 (1.25)
Change in victimization among victims without defenders, *M* (SD)	−0.44 (1.17)	−0.48 (1.30)
Baseline popularity (proportion) among all participants, *M* (SD)	0.15 (0.17)	0.14 (0.16)
Baseline popularity (proportion) among all defenders, *M* (SD)	0.16 (0.17)	0.17 (0.17)
Baseline popularity (proportion) among all victims, *M* (SD)	0.14 (0.16)	0.14 (0.16)

*Note*: *N* = 93 classes: initial sample for which social network analysis was computed.

See the Supporting Information S5 (Tables S5.1 and S5.2) for a comparison of the descriptive statistics of the initial sample of 93 classes and the sample of 44 classes for which model 1 converged.

### Uniplex network, covariate and behavior effects

To account for general tendencies in tie formation, both structural and covariate network effects were included in the model. Table 4 presents the results of the meta‐analyses conducted across classes. The *outdegree* parameter was negative (Est. = −2.62, SE = 0.20, OR = 0.07, *p* < .001), indicating that defending nominations were relatively sparse. The *outdegree activity* parameter was positive (Est. = 0.55, SE = 0.07, OR = 1.73, *p* < .001), indicating that students who were already defended were more likely to be defended by additional students over time. The positive *same‐gender* parameter shows that students were more likely to be defended by students of the same gender (Est. = 0.44, SE = 0.11, OR = 1.55, *p* < .001). To account for individual‐level behavioral tendencies over time, the linear and quadratic shape parameters were estimated. The *linear shape effect* (Est. = −0.99, SE = 0.08, OR = 0.37, *p* < .001) indicates an overall tendency toward lower victimization scores, while the *quadratic shape effect* (Est. = 0.26, SE = 0.05, OR = 1.30, *p* < .001) reflects a self‐reinforcing pattern in victimization.

**TABLE 4:** Results of meta‐analyses across classes.

	Model 1			Model 2a			Model 2b			Model 2c		
	*N*	Est.	SE	*N*	Est.	SE	*N*	Est.	SE	*N*	Est.	SE
Defending dynamics (structural & covariate network effects)												
Outdegree (density)	33	−2.62	0.20***	23	−2.45	0.23***	36	−2.63	0.22***	17	−2.46	0.29***
Outdegree activity	40	0.55	0.07***	25	0.51	0.08***	30	0.52	0.08***	19	0.47	0.10***
Same gender	38	0.44	0.11***	28	0.31	0.12**	31	0.38	0.12**	21	0.41	0.14**
Victimization dynamics (behavior effects)												
Linear	41	−0.99	0.08***	27	−0.95	0.11***	32	−1.02	0.10***	21	−0.98	0.13***
Quadratic	39	0.26	0.05***	26	0.26	0.06***	32	0.25	0.06***	20	0.25	0.07***
Defending (network) → victimization (behavior)												
D outdegree → V	38	0.04	0.04	25	0.04	0.06	32	0.05	0.05	18	0.06	0.06
D outdegree × baseline popularity defender → V				22	−0.01	0.10				15	−0.02	0.12
D outdegree × baseline popularity victim → V							28	−0.27	0.41	15	−0.51	0.60

*Note*: Fixed rates of change effects were omitted from the table. **p < .01; ***p < .001 (two‐tailed tests). A model including the reciprocity parameter (Est = 1.02, SE = 0.35, *p* < .001) yielded comparable results for other network parameters; but the reciprocity parameter caused significant convergence issues. Therefore, we only presented models in which the reciprocity parameter was omitted. See the Supporting Information S3 for details.

Abbreviations: D, defending (network); *N*, number of classes included in the meta‐analysis for this parameter; *V* = victimization (behavior).

### Network‐behavior dynamics

Effects modeling behavioral evolution were included in the models to test our hypotheses and conduct exploratory analyses. Results from model 1 indicate that outdegree in the defending network did not significantly predict changes in the frequency of victimization over time (Est. = 0.04, SE = 0.04, OR = 1.04, *p* = .319). This finding contradicts our hypothesis that nominating more defenders is related to a greater decrease in victimization frequency over time. Notably, the direction of the parameter estimate was opposite to what we had expected.

In the second set of models, we examined potential moderators through interaction terms. Model 2a tested whether the effect of defending outdegree on victimization was moderated by the defenders' baseline popularity. The interaction was not significant (Est. = −0.01, SE = 0.10, OR = 1.01, *p* = .953). Additional analyses, in which total defender popularity was modeled as a victim attribute, allowing for a larger number of classes to be included, yielded consistent results: the interaction remained non‐significant. See the Supporting Information S6 (Table S6.1) for details. Model 2b tested whether the victim's popularity at baseline moderated the effect of defending outdegree on victimization. Again, no significant interaction was found (Est. = −0.27, SE = 0.41, OR = 0.76, *p* = .518). Finally, in Model 2c, where both interaction terms were included simultaneously, the results remained consistent: neither the interaction with defenders' baseline popularity (Est. = −0.02, SE = 0.12, OR = 0.98, *p* = .833) nor with the victim's baseline popularity (Est. = −0.51, SE = 0.60, OR = 0.60, *p* = .396) reached statistical significance.

### Grade effects

To examine whether the parameters of interest differed by grade, grade level was included as a multilevel continuous predictor in the meta‐analyses. As shown in the Supporting Information S7 (Table S7.1), grade did not predict any of these parameters, indicating that there were no differences between grades in the *defending outdegree → victimization effect*, nor in its interaction with the defenders' or victim's baseline popularity.

### Goodness of fit

Overall, the models demonstrated acceptable goodness of fit for nearly all inspected indices. In most cases, the observed data fell within the simulated distribution, as reflected in the violin plots and supported by *p*‐values greater than .05. Exceptions were limited: the *p*‐value for the *indegree* distribution was below .05 in 3 classes in model 1 (model 2a: 1 class; model 2b: 2 classes; model 2c: 0 classes). For the *outdegree* distribution, *p*‐values were below .05 in 2 classes in model 1 (model 2a: 2 classes; model 2b: 1 class; model 2c: 1 class). For the triad census distribution, *p*‐values were below .05 for 5 classes in model 1 (model 2a: 1 class; model 2b: 4 classes; model 2c: 0 classes). Throughout models, there was one class for which the goodness of fit statistics was missing. The full goodness of fit results, including all *p*‐values and violin plots, can be found in the “gof_summary” HTML files available for each of the models on our OSF project page.

## DISCUSSION

Many school‐based interventions target peer bystanders, encouraging them to actively defend against bullying (Gaffney et al., 2021). However, recent theoretical and empirical studies have questioned the effectiveness of this approach in reducing victimization (Healy, 2020; Laninga‐Wijnen et al., 2023). To address these concerns, we employed a longitudinal social network approach to examine whether nominating more defenders would predict changes in victimization over time among (early) adolescents and whether this effect would be moderated by the defender's and victim's baseline popularity.

Contrary to our expectations, nominating more defenders did not predict a reduction in the subsequent frequency of victimization. It is important to note that null hypothesis significance testing does not permit conclusions about the truth of the null hypothesis; it only indicates whether the observed data are unlikely under the assumption that the null is true (Harms & Lakens, 2018). Therefore, we cannot definitely conclude that defending has no effect on subsequent victimization; we can only conclude that we did not find evidence of such an effect in this study. Descriptive patterns in our data likewise provide no indication that defending contributes to reductions in victimization. More than two‐thirds of victimized students nominated at least one defender, indicating that defending was relatively common. In the first period, victims who did not nominate any defender showed slightly greater reductions in victimization than those who nominated at least one defender, while in the second period, the trend reversed. These descriptive trends do not suggest a consistent advantage for victims with defenders, which reinforces the conclusion that the presence of defenders alone may not reduce victimization over time.

We hypothesized that the popularity of defenders would exacerbate the effects of defending because popular students typically have better social skills and more social power among their peers (e.g., Dawes & Norwalk, 2022). However, we did not find evidence to support this hypothesis. This finding aligns with previous work indicating that popularity norms for defending do not moderate the association between being defended and later victimization frequency (Laninga‐Wijnen et al., 2023), although that study did not assess the popularity of specific defenders in relation to individual victims. From a statistical perspective, convergence issues limited the number of classrooms included in the interaction models, which may have reduced our ability to detect subtle moderating effects. From a theoretical perspective, popularity may not consistently predict defending effectiveness because popular students are heterogeneous: some are known for prosocial behavior, some gain status through aggression (De Bruyn & Cillessen, 2006), and some pursue popularity by combining aggressive and prosocial behaviors (Hawley, 2003). If defenders are perceived as aggressive, their actions may lack credibility. Therefore, it is possible that only defending by consistently prosocial popular students influences bullying dynamics. Future research should explore how the social reputation and perceived intent of defenders impact the effectiveness of defending in reducing victimization.

Furthermore, we found no evidence that nominating more defenders affects changes in victimization based on the victim's baseline popularity. Once again, convergence issues may have limited our ability to detect subtle moderation patterns. From a theoretical perspective, we reasoned that targeting a popular victim who is defended might pose a greater perceived social risk to the bullies, which could make defending more effective for popular victims. However, defending popular victims could backfire if it draws attention to their victim status, thereby undermining their social image (Healy, 2020). Thus, defending may fail to reduce victimization for both popular and unpopular students.

The finding that defending does not reduce subsequent victimization is consistent with several prior longitudinal studies on this topic. For instance, one study found no difference in victimization trajectories between defended and non‐defended victims among elementary school students in the Netherlands (Laninga‐Wijnen et al., 2023). Another study of Dutch elementary‐school students found that victims without a designated peer support group experienced a decrease in victimization over the school year, whereas those with a support group did not (van der Ploeg et al., 2016). Another very recent study, which used a data set partially similar to the one used in our study, found no evidence that victim‐oriented or bully‐oriented defending reduced specific types of victimization over time (Laninga‐Wijnen, Yanagida, & Garandeau, 2025). Our social network study builds on this prior work by examining the role of defenders' and victims' popularity in the effectiveness of defending while considering the interdependent structure of victim–defender networks.

We found no evidence that the effect of having more defenders on victimization depends on the defender's or victim's baseline popularity. While this may suggest that defending is generally ineffective in reducing subsequent victimization, it is also possible that other individual or contextual factors moderate the effectiveness of defending, protecting some students while increasing risk for others. Several reasons have been hypothesized as to why defending victims of bullying may have iatrogenic effects (Healy, 2020): First, when others defend them, victims may feel disempowered to stand up for themselves, which may facilitate bullies to continue harassing this victim. Second, defending may provoke retaliation from bullies. Third, defending may demonstrate the victim's vulnerable status in the peer group, which may attract the attention of potential new perpetrators. If defending yields protective effects for some students and adverse effects for others, these effects may cancel each other out, which could explain the overall null effect observed. These results underscore the need for further research on the impact of defending behavior. While considerable research has focused on the antecedents of defending, far less is known about whether and under which conditions it effectively reduces victimization. Clarifying these circumstances is crucial for informing the design of interventions that rely on peer defending as a core mechanism for bullying prevention.

One key factor that may determine the effectiveness of defending is *how* students defend. Previous studies have identified various ways to classify different types of defending. For instance, they have distinguished between victim‐oriented and bully‐oriented defending (Reijntjes et al., 2016), and between direct (aggressive and solution‐focused) and indirect (comforting and reporting to an authority) defending (Lambe & Craig, 2020). However, even within these categories, the way defending is enacted can vary widely in tone, formulation, assertiveness, and perceived intent – factors that can influence its effectiveness or render it counterproductive. For example, aggressive defending may escalate conflict (Lambe & Craig, 2020), which supports the argument that defending can provoke retaliation from bullies (Healy, 2020). Similarly, if defending highlights a victim's inability to cope independently, it may reinforce their vulnerable position and sustain victimization (Healy, 2020). Some anti‐bullying programs, such as KiVa, provide guidelines like encouraging students to seek help from others or involve a teacher (Salmivalli et al., 2010). Despite this, students often receive only general messages encouraging them to intervene, without clear instructions on how to do so effectively (Casey et al., 2017). Future research should examine the variation in how defending is enacted to inform interventions that provide clear, evidence‐based recommendations for *effective defending strategies*.

The effectiveness of defending may also depend on the type of bullying targeted. Direct bullying, such as verbal or physical aggression, is more visible than indirect (or relational) bullying, such as exclusion or rumor spreading (Salmivalli et al., 2021). Indirect bullying is more subtle and embedded in social dynamics, making it harder to detect and disrupt through defending. Therefore, defending may be more effective against direct forms of bullying. Future research should examine whether the form of bullying moderates the effectiveness of defending.

Furthermore, the effectiveness of defending may depend on the broader social context, particularly the presence of an audience and how peers respond to the act of defending. If defending occurs in a setting where peers endorse positive social norms (e.g., where defending is valued or expected), it may be more successful in reducing bullying. Although previous studies found that classroom‐level descriptive and popularity norms did not affect the effectiveness of defending (Laninga‐Wijnen et al., 2023), other social norms, such as those within friendship groups or signaled by teachers, may be more influential.

Finally, individual characteristics other than popularity may moderate the effectiveness of defending. For defenders, leadership status may be more decisive than popularity. Prior research has identified distinct leadership profiles, including positive leaders, negative leaders, and non‐popular leaders (Dong et al., 2023). Future studies could examine whether these profiles moderate the effectiveness of defending. In addition, defenders' prior involvement in bullying may affect how their defending is perceived, as those with a history of bullying may provoke skepticism from peers. On the victim side, defending may be less effective for those with a history of chronic victimization, as their established social position within the peer group may be more resistant to change (Goldbaum et al., 2003). Ultimately, the success of defending may depend not only on individual traits but also on particular combinations, and future research should aim to uncover this “recipe” for effective defending.

### Strengths and limitations

A key strength of this study is its relational perspective on defending. Using a longitudinal social network approach allowed us to capture the dynamic interplay between victims and their defenders and to test the moderating role of popularity as an individual attribute of both defenders and victims. This approach enabled us to model the interdependent nature of defending relationships, where multiple victims can be supported by the same defender, and the same victim can be defended by multiple peers. In addition, the models explicitly accounted for the structural tendencies to nominate defenders and the constraint that only victims can be defended.

At the same time, the network‐based methodology presented certain challenges. A substantial portion of the initial sample had to be excluded due to model non‐convergence. Importantly, we had anticipated this issue and explicitly addressed it in our preregistration. Supplementary analyses (see Table 1 and the Supporting Information S4) indicated that there was a tendency for convergence to be less likely in sparse networks and in networks with very low levels of victimization. Moreover, models were less likely to converge for smaller classes. Given the methodological requirements of our longitudinal social network approach, studying defending is only feasible in contexts where victimization occurs. Classes with minimal victimization had to be excluded, introducing some degree of selectivity. Theoretically, classes could also be excluded due to low levels of defending despite substantial victimization, which would be a more problematic exclusion. However, our additional analyses did not support this pattern, suggesting that the final sample retained its relevance for the research question.

Nevertheless, the final sample primarily consisted of larger classrooms with moderate levels of victimization and defending. This systematic selection warrants caution when generalizing the findings to small classrooms or classrooms where the overall levels of victimization and/or defending are low. Unfortunately, models testing the interaction effects were even more prone to non‐convergence, which reduced the available sample size for moderation analyses. This limitation constrains the generalizability of these findings and indicates that the results concerning the moderating roles of defender and victim popularity should be viewed as preliminary and interpreted in light of these methodological constraints.

Importantly, the finding that having more defenders was unrelated to subsequent reductions in victimization is consistent with results from multiple studies applying traditional regression models (e.g., Laninga‐Wijnen et al., 2023). This finding also corresponds with a recent study on a partially overlapping sample that found that neither victim‐oriented nor bully‐oriented types of defending reduced a latent factor consisting of five specific types of victimization (Laninga‐Wijnen, Yanagida, & Garandeau, 2025). Our study builds upon this prior work by examining the potential moderating effects of defenders' and victims' popularity while accounting for the interdependent structure of victim‐defending ties.

This study was conducted in Finland, a country with high academic achievement and educational equity (Ustyn & Eryilmas, 2018). The prevalence of victimization in our sample (approximately 30%) is consistent with prevalence rates found in previous research, which showed that Finland tends to have lower bullying rates than many other countries in global comparisons (Johansson et al., 2022). The defending networks in the current data were relatively sparse compared to those reported in existing studies on defending networks, most of which were conducted with a sample from Dutch elementary schools (e.g., Huitsing et al., 2014). These contextual factors may shape bullying dynamics and limit the generalizability of our findings to countries with different educational systems. Future research should replicate this study in various cultural and educational contexts to assess the broader applicability of the results.

### Implications and future directions

Importantly, the effectiveness of defending can be evaluated based on several approaches. One approach is to examine whether defending reduces the overall prevalence of bullying at the group level. Another approach is to assess whether it buffers the negative psychosocial effects of victimization for individual victims. A third approach, which we adopted in this study, focuses on whether being defended leads to a reduction in the frequency of victimization over time among those who identify as victims. Although our findings suggest that defending does not reduce subsequent victimization, it may still be effective in achieving other outcomes. Prior research has shown that defending is associated with lower levels of bullying at the classroom level (Salmivalli et al., 2011) and with improved psychosocial adjustment among victims, including greater feelings of belonging (Laninga‐Wijnen et al., 2023), higher self‐esteem (Sainio et al., 2011), and better emotional well‐being (Ma & Chen, 2019). We therefore do not argue against encouraging students to defend their peers. Rather, our findings suggest the need for a better understanding of why defending may fall short in reducing victimization itself and the need to explore additional or complementary strategies that could enhance the effectiveness of bullying prevention and intervention. Below, we outline several areas where additional efforts could strengthen the overall effectiveness of anti‐bullying initiatives:

First, popularity is a key motive in bullying, but it is not the only one: a review identified several additional motives such as revenge, belonging, and entertainment (Sanders et al., 2021). These diverse motives suggest that defending may not affect all bullies in the same way. An important direction for future research is to examine whether being confronted with defending reduces bullying over time from the perspective of the perpetrators. Distinct subtypes of bullies have been identified (popular‐liked bullies; popular‐rejected bully–victims, and bully–victims; Turunen et al., 2024), who may vary in their responsiveness to defending. For instance, popular‐liked bullies often hold strong status goals (Košir et al., 2022) and therefore may be responsive to defending, especially when it threatens their social status. In contrast, bully–victims may perceive defending as provocation and respond with retaliation (Peeters et al., 2010). Future research should investigate the mechanisms that explain why defending deters some bullies but not others.

Second, being defended does not necessarily alter how victims are perceived within the social group. If peers continue to view victims as weak or socially isolated, defending may offer limited long‐term protection. Moreover, defending is not always an available response: findings from a daily diary study show that in 64% of bullying incidents, bystanders are absent (Laninga‐Wijnen et al., 2024). This highlights the need to equip victims with other forms of support, including strategies for self‐protection. Peer‐based approaches should therefore be complemented by interventions aimed at empowering victims themselves. A review of meta‐analyses identified self‐oriented personal competencies, like self‐esteem and self‐related cognitions, as among the strongest protective factors against face‐to‐face victimization (Zych et al., 2019). Strengthening these individual competencies may help potential future victims respond more effectively to social challenges and reduce their vulnerability over time.

Third, it may not be realistic to expect peers alone to stop bullying. Teachers and other school staff must take active responsibility for addressing bullying when it occurs (Veenstra et al., 2014), and the role of targeted interventions deserves greater attention. Much of the existing research has focused on prevention, while comparatively little is known about effective strategies for intervening after bullying has already taken place (Laninga‐Wijnen, Huisman, et al., 2025). Short‐term outcomes from targeted teacher‐led interventions appear promising. In a recent study, 88% of victims reported a reduction or cessation of victimization following targeted intervention through teachers, with approaches that combined empathy‐raising and explicit condemnation of the behavior proving most effective (Laninga‐Wijnen, Huisman, et al., 2025). Teachers themselves recognize the limits of peer intervention: teacher reports obtained from the SOLID project show that 85% of teachers think that peer defending can only occasionally stop bullying. These findings underscore the importance of implementing teacher‐led targeted interventions as a core element of bullying intervention. Moreover, research on the effectiveness, mechanisms of change, and optimal strategies for such interventions should be expanded and further prioritized.

Ultimately, there is likely no single solution to the complex problem of bullying. Effective intervention may require a multifaceted approach that addresses individual, peer, and school levels. Broadening current strategies by exploring new directions, testing innovative approaches, and combining efforts across different domains may offer the best chance of creating lasting change in reducing bullying and its negative consequences.

## CONCLUSION

Using a longitudinal social network approach, this study examined whether nominating more defenders reduces subsequent victimization among (early) adolescents and whether this effect is moderated by the popularity of the defender and the victim. Contrary to expectations, nominating more defenders did not predict victimization over time, and no moderating role of the popularity of defenders and victims was detected. These findings contribute to growing evidence that the effectiveness of defending in reducing victimization may be more limited than previously assumed. Defending alone may not be sufficient to disrupt the broader social processes that sustain bullying.

## AUTHOR CONTRIBUTIONS

S.R.: conceptualization, data curation, methodology, formal analysis, writing – original draft. Mv.Z.: conceptualization, writing – review and editing. R.V.: conceptualization, methodology, writing – review and editing. L.L.W.: conceptualization, methodology, investigation, project administration, funding acquisition, supervision, writing – review and editing. All authors read and approved the final manuscript.

## FUNDING INFORMATION

The SOLID project has been funded with the Postdoctoral Researcher Grant (No. 349560) from the Academy of Finland, awarded to Lydia Laninga‐Wijnen. There was no role of the funding source in design, analysis, writing of the report.

## CONFLICT OF INTEREST STATEMENT

The authors report no conflict of interest.

## ETHICAL APPROVAL

The study has been approved by the Ethical Board of the University of Turku in Finland (November 2021, reference number 53/2021).

## PREREGISTRATION

The study design, sampling, and materials were preregistered at https://osf.io/nghwm. The study's hypotheses and analyses presented in this paper were preregistered at https://osf.io/sv3hk.

## DECLARATION OF GENERATIVE AI AND AI‐ASSISTED TECHNOLOGIES IN THE WRITING PROCESS

During the preparation of this work, the authors used ChatGPT to revise and improve the wording of selected sections of the manuscript. After using this tool, the authors reviewed and edited the content as needed and take full responsibility for the content of the publication.

## INFORMED CONSENT

Informed consent was obtained from all individual participants included in the study, as well as from their legal guardians for (1) participating in the study and (2) archiving data.

## Supporting information


Data S1:

## Data Availability

This study's analysis code and results output are available at https://osf.io/tvrcx/. Data are available upon request from the last author and data of students with consent for archiving will be publicly available after project completion (end of 2025).
