# Supplementary material for: The role of defenders' and victims' popularity in the effectiveness of defending in bullying interactions: A longitudinal social network study
Source: J Res Adolesc. 2025 Dec 30;36(1):e70126. doi: 10.1111/jora.70126 (PMC12754034; doi:10.1111/jora.70126)
Supplement: Supplementary file 1 — Data S1: [file JORA-36-0-s001.pdf]

Supplemental materials to:

The Role of Defenders' and Victims' Popularity in the Effectiveness of Defending in Bullying Interactions - A Longitudinal Social Network Study

Table of content:

S1: Deviations From the Preregistration……………………………………………………..…2

S2: Data-Driven Specification of Structural and Covariate Effects………………………..…..4

S3: Additional Analyses Model 1: Including the Reciprocity Parameter……..………………6

Table S3.1. Results of the meta-analysis across classes for model 1 with reciprocity parameter……………………………………………………………………………7

S4: Examination of Class Characteristics Associated With Model Convergence…………...…8

S5: Descriptive Statistics of the Initial Sample and the Final Sample of Model 1…..………13

Table S5.1. Descriptive statistics of defending networks per wave for the initial sample and the final sample of model 1……………………………………..…………..14

Table S5.2. Descriptive statistics of victimization, popularity defending per wave for the initial sample and the final sample of model 1……………..…………………..15

S6: Additional Analyses Model 2a: Coding Defender Status as a Victim Attribute…………17

Table S6.1. Results of the meta-analysis across classes for model 2a with defender status as a victim attribute……………………………………………………….18

S7: Grade Effects…………………………………..………………………………19

Table S7.1 Grade effects on the parameters of interest………………………………19

References………………………………………………………………………………….20

S1: Deviations From the Preregistration

Several deviations from the preregistration were necessary for the following reasons:

(1) Inclusion criteria: The preregistration stated that we would include only those classes with a participation rate of at least 80% in at least one wave. However, overall participation rates were lower than anticipated. To avoid excluding a substantial number of classes in which model convergence still appeared feasible, we lowered the inclusion threshold to 70% in at least one wave.

(2) Coding of the popularity covariate: The preregistration stated that the proportion of received popularity nominations would be calculated by dividing the number of nominations each student received by the total number of students in the class. Upon closer consideration, we refined this approach: the denominator was limited to students who actually responded to the nomination item, as only they could serve as potential nominators. The original description was imprecise, as including non-respondents in the denominator would underestimate students' relative popularity.

(3) Coding of the defending network: We initially stated that we would transpose the defending network matrices, resulting in ties representing *defender → victim* relationships rather than *victim → defender* relationships. However, when specifying effects for the second model, we realized that the *ego* (i.e., the individual at the center of the analysis) must be the victim, as our outcome of interest was the severity of victimization of the victim. Consequently, we did not transpose the matrices, thereby preserving the *victim → defender* direction of ties.

(4) Specification of network effects: As a result of the adjustment described in (3), we also modified the specification of the network effects. Specifically, to test our primary hypothesis that being defended predicts lower subsequent victimization, we included the *outdegree* effect from the defending network rather than the originally preregistered *indegree* effect. Additionally, what would have been the *inPopSqrt* effect

in the transposed matrices corresponds to the *outActSqrt* effect in the current specification.

(5) Three-way interaction: The preregistration stated that we would explore whether there is a three-way interaction between the longitudinal effect of being defended on the frequency of victimization and the baseline status of the defender and victim. However, model stability declined as additional effects where included, and the model failed to converge in most classes when the three-way interaction was added. Given these convergence issues, we did not report this analysis in the manuscript.

S2: Data-Driven Specification of Structural and Covariate Effects

As noted in S1, the initial analyses for Model 1 were conducted on the transposed defending network matrices, consistent with the preregistration. The following steps outline our iterative, data-driven model refinement process:

We began by specifying all effects as outlined in the preregistration. However, the model failed to converge in any class under this specification. Given the sparsity of the defending networks, we had to substantially simplify the model. Through an iterative process of trial and error, primarily removing effects, we identified an initial reduced specification that allowed convergence in 14 classes. This initial reduced model included the structural effects *inPopSqrt* and *inActSqrt*, along with gender *egoX, altX,* and *sameX* as covariate effects. As the meta-analysis of these initial results indicated that the *inActSqrt* and gender *egoX* and *altX* effects were non-significant, we excluded them from the subsequent model. This model included only the *inPopSqrt* effect as a structural effect and the same gender (*sameX*) effect as a covariate effect. It converged in 25 classes. To further improve convergence, we explored several additional modifications, none of which resulted in more converged classes:

- Adding the *outIso* effect, following previous work on defending networks (Huitsing et al., 2014)
- Including the *outActSqrt* effect
- Conducting period-wise analyses (e.g., separately analyzing transitions from Wave 1 → Wave 2 and Wave 2 → Wave 3)

We then identified a conceptual limitation in our specification: we had only set allowOnly = FALSE for the defending network, not for the victimization network. Allowing two-sided simulations for victimization (even when changes were observed only in one direction) was more theoretically appropriate. Implementing this change resulted in 22 converged classes, fewer than our prior best solution, but better aligned with our theoretical framework.

Additional modifications explored included:

- Removing the quadratic shape effect (no improvement)
- Recoding the victimization variable by collapsing categories 3 and 4 (yielded one additional converged class but led to information loss about changes in victimization; therefore, the original coding was retained)
- Fixing the defending rate parameters to values from prior meta-analysis across classes, followed by fixing the victimization rate parameters, and setting the initial values of all other parameters based on previous meta-analyzed estimates. This improved convergence.

Throughout this process, our key finding remained robust: the *indegree* effect on victimization was consistently non-significant across all model specifications.

When progressing to Model 2, which included interaction effects, we encountered a problem due to the transposition of the defending network. Because we aimed to predict victimization of the victim, the *ego* needed to represent the victim to correctly specify interaction effects. As a result, we reverted to using non-transposed matrices, where ties represent *victim → defender* relationships. This required updating the effect specification accordingly, replacing *inPopSqrt* with *outActSqrt*, and *indeg* with *outdeg*.

In the first run using this model, 22 classes converged. We then set the initial parameter values using the estimates from the meta-analysis, which increased the number of converged classes to 24. Next, we fixed the rate parameters for the defending network to the values obtained in this previous model. Now the model converged for 27 classes. Finally, by fixing the rate parameters to values obtained from the most recent model, convergence improved further, reaching 44 classes.

S3: Additional Analyses Model 1: Including the Reciprocity Parameter

Although the reciprocity effect - representing the tendency for mutual nominations - is typically included by default in RSiena models, we initially excluded it due to convergence problems observed during preliminary model runs. Descriptive statistics indicated that among all non-missing dyads where reciprocity could be measured, 35% of ties were reciprocated at wave 1, 34% at wave 2, and only 15% at wave 3. This sharp drop in reciprocity at wave 3 may have contributed to estimation difficulties. In addition, the large standard errors for the reciprocity estimates (ranging from .30 to .40 across waves) suggest substantial between-class variability, which could also have impeded model convergence.

To assess the robustness of our findings, we re-estimated model 1 including the reciprocity parameter across all classes. With this specification, only 14 classes reached convergence, compared to 22 classes in a comparable model without the reciprocity effect (i.e., without fixed rate parameters and specified initial values, see S2). The results of this model are reported in Table S3.1. While the reciprocity parameter itself was statistically significant, the parameter estimates for all other effects closely mirrored those found in model 1 as reported in the main manuscript. This suggests that excluding reciprocity did not meaningfully alter the main conclusions of the study, but the inclusion of the effect clearly reduced the number of analyzable cases due to convergence constraints.

Table S3.1

Results of the meta-analysis across classes for model 1 with reciprocity parameter

	N	Est.	*SE*
Structural & covariate network effects			
Outdegree (density)	12	-2.80	0.24***
Reciprocity	10	1.02	0.35**
Outdegree Activity	13	0.49	0.07***
Same sex	13	0.38	0.17*
Behavior effects			
Linear	14	-1.03	0.14***
Quadratic	13	0.21	0.05***
Network → behavior			
D indegree → V	13	0.06	0.04

Note. Rate of change effects were omitted from the table. *N* = number of classes included in the meta-analysis for this parameter. * $p < .05$; ** $p < .01$; *** $p < .001$ (two-tailed tests).

S4: Examination of Class Characteristics Associated With Model Convergence

Social network analyses were conducted in RSiena for our sample of 93 classes. In these analyses, network parameters are only interpretable when the model successfully converges, defined as the maximum overall convergence ratio remaining below .25 (Ripley et al., 2024). In Model 1, convergence was achieved for 44 out of 93 classes (47.31%). It was expected that the models would not converge for all classes, as defending networks tend to be sparse. As outlined in our preregistration, several factors can contribute to the potential sparsity of defending networks:

First, low response rates can contribute to network sparsity. However, classes with response rates below 70% in at least one data collection wave were already excluded during an earlier stage of data processing. Second, low levels of victimization, reflected by a high proportion of structural zeros – impossible ties arising because only victimized students could nominate defenders – may have impeded convergence. Across classes, the proportion of structural zeros ranged from 21% to 90% in Wave 1, from 33% to 92% in Wave 2, and from 48% to 93% in Wave 3. Third, limited defending behavior itself, indicated by low outdegrees, could have contributed to convergence difficulties. RSiena-based models require sufficient network density for reliable parameter estimation, with average degrees below 2 often associated with convergence issues (Ripley et al., 2024). In our data, defending network average degrees were overall very low, and varied considerably across classes, ranging from 0.00 to 2.21 in Wave 1, 0.00 to 1.43 in Wave 2, and 0.00 to 1.87 in Wave 3.

To examine factors associated with model convergence, we conducted logistic regressions, testing network density (outdegrees; for each wave) and the proportion of structural zeros (for each wave) as predictors in separate models. The results are displayed in Table 1 in the main manuscript. We also visualized these associations, plotting the predictor on the x-axis and convergence status (1 = converged, 0 = non-converged) on the y-axis, with each dot representing one of the 93 classes. Although there was a tendency for higher

proportions of structural zeros (indicating lower victimization) to be associated with a reduced likelihood of convergence, this trend was not statistically significant for any wave. Regarding network density, there was a tendency for classes with lower density to be associated with a lower likelihood of convergence throughout all waves, but only the T3 outdegree reached statistical significance. Neither T1 nor T2 outdegrees were significant predictors. The non-significance of these logistic regressions could be due to limited power given the small sample size of 93 classes.

Given the methodological requirements of the current approach, studying defending is only feasible in contexts where victimization occurs. Classes with minimal victimization had to be excluded, introducing some degree of selectivity. Theoretically, classes could also be excluded due to low levels of defending despite substantial victimization – an exclusion that would be more problematic. To investigate this, we identified non-converged classes characterized by both low victimization (proportion of structural zeros one standard deviation below the mean) and low defending (density one standard deviation below the mean). None of the 49 non-converged classes in Model 1 met these criteria across any wave, suggesting that model non-convergence was not systematically driven by a lack of defending in classes with high victimization.

Given that the expected class characteristics were only partially associated with model convergence, we explored additional factors that might account for convergence. Specifically, we examined class size and Jaccard indices (for each period) as potential predictors of convergence. We conducted additional logistic regressions, with results again presented in Table 1 of the main manuscript. The analyses revealed that Jaccard indices did not significantly predict convergence, while class size showed a positive association: models were more likely to converge in larger classes.

Plots showing the probability of convergence depending on characteristics of the defending network

T1 proportion of structural zeros in the defending network

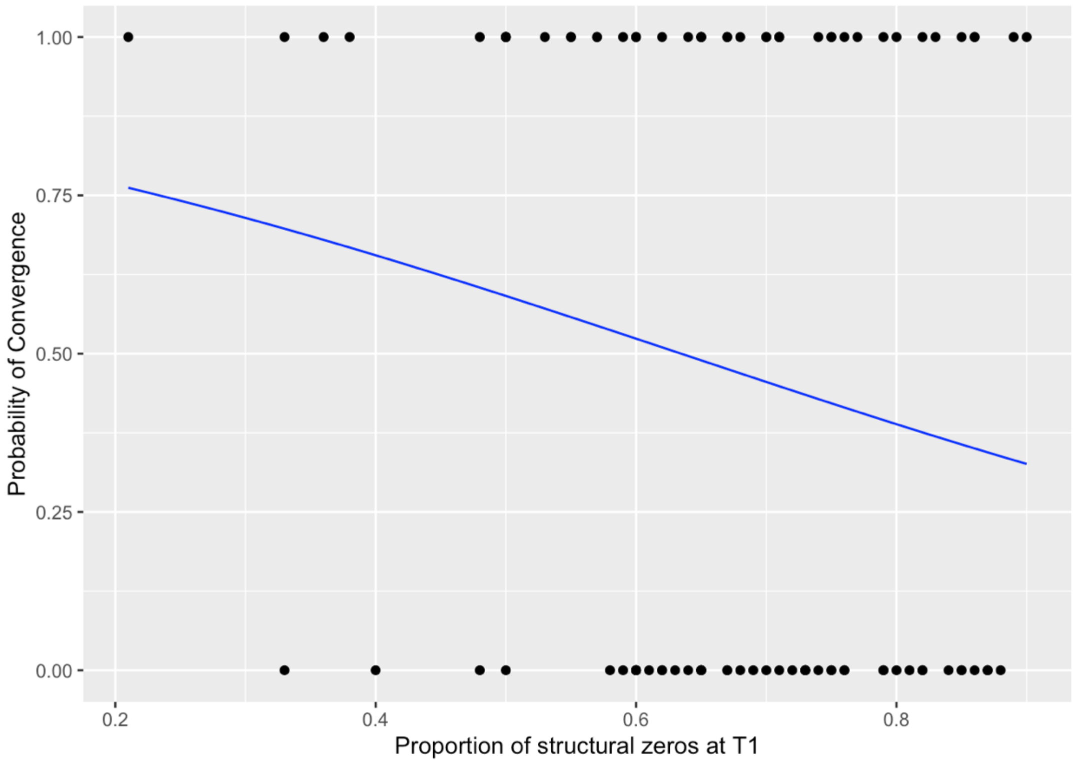

T1 density in the defending network

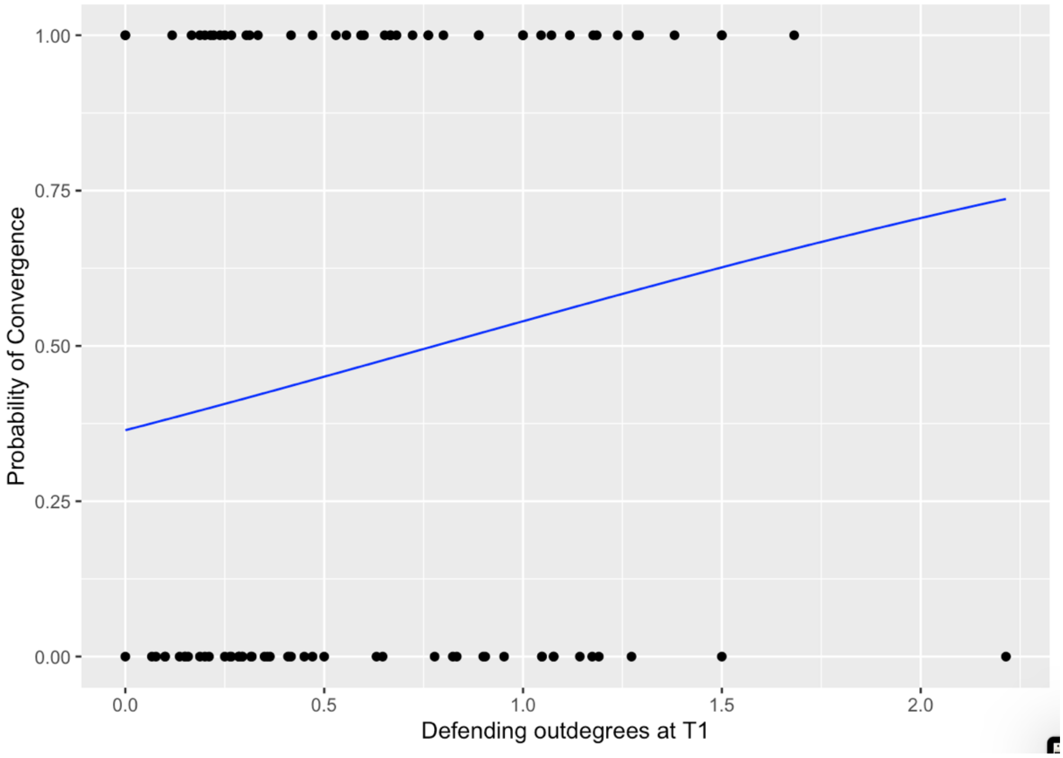

T2 proportion of structural zeros in the defending network

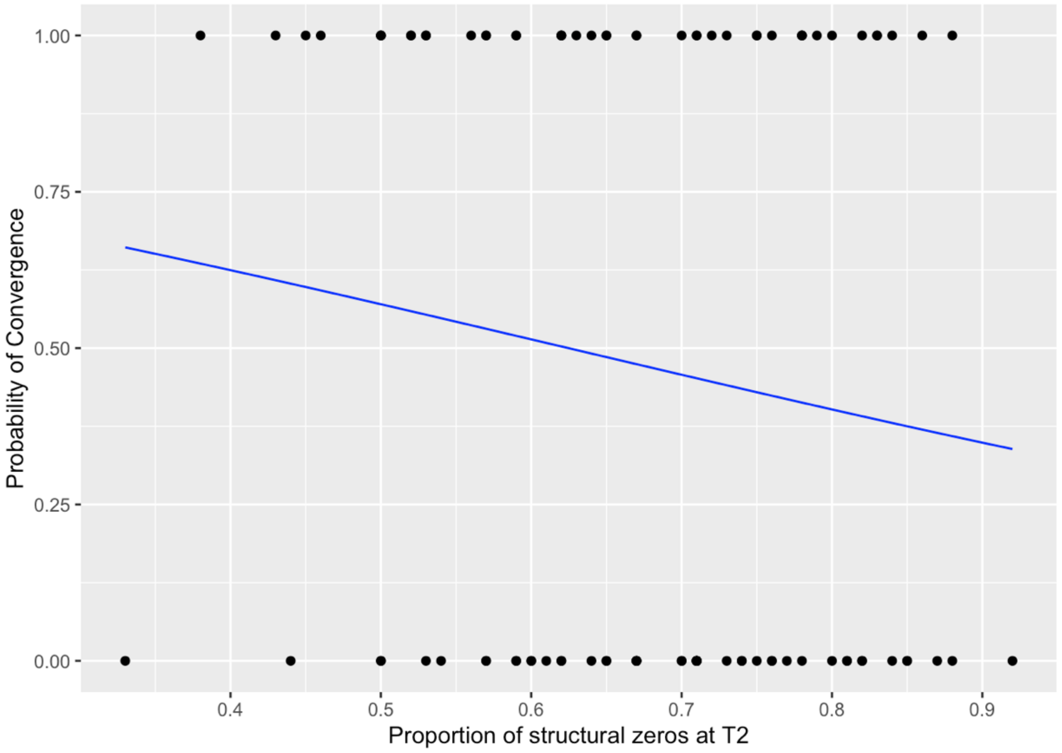

T2 density in the defending network

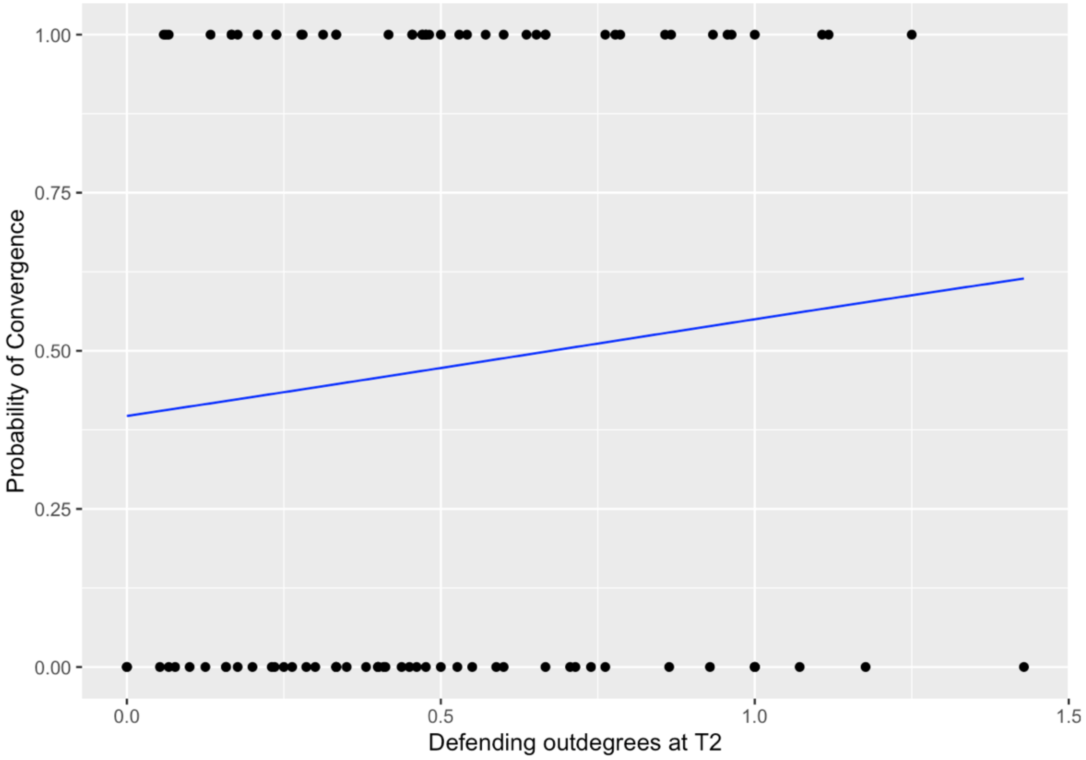

T3 proportion of structural zeros in the defending network

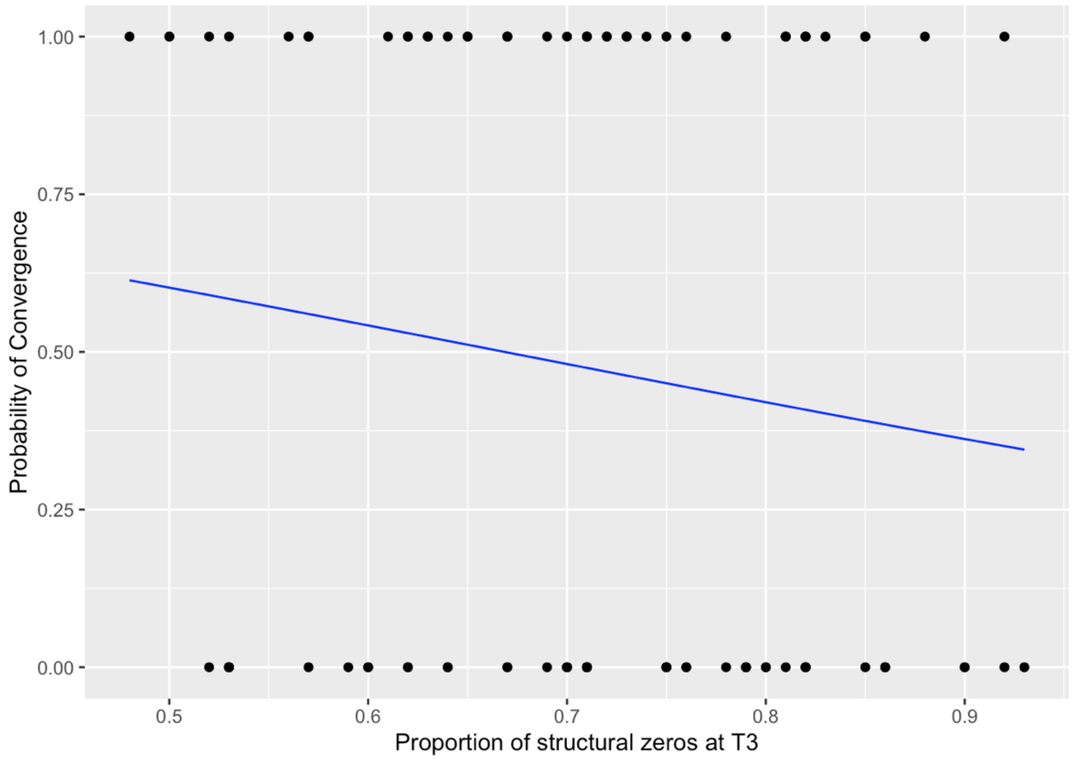

T3 density in the defending network

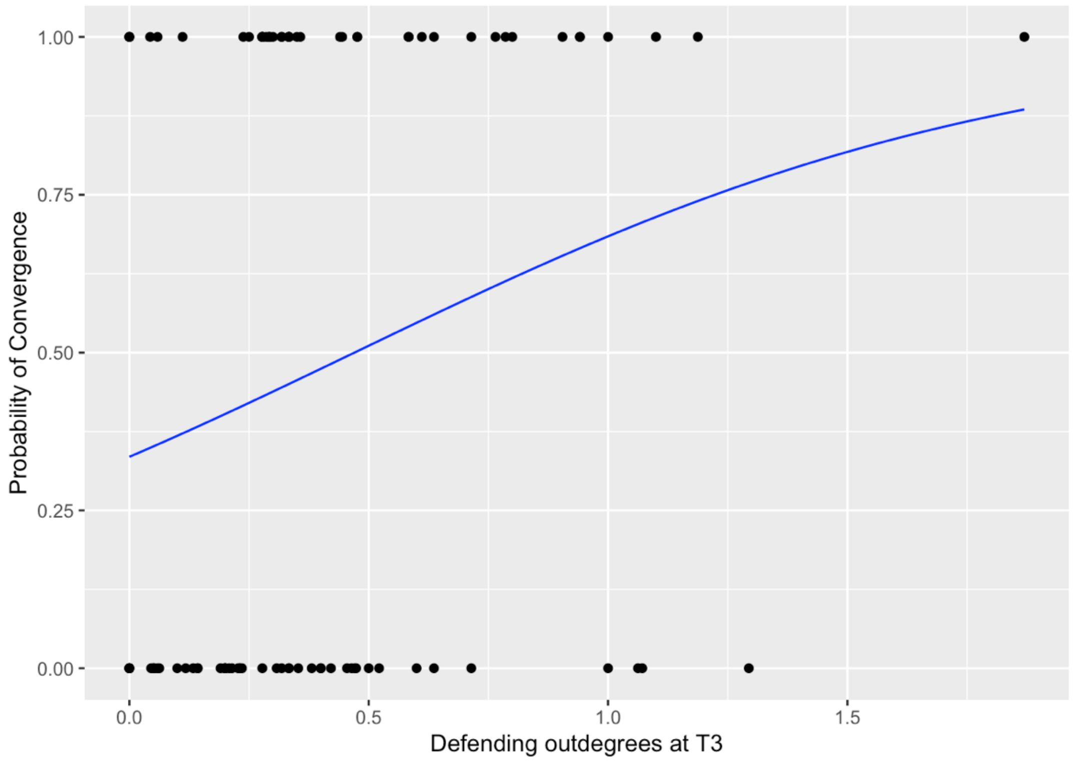

S5: Descriptive Statistics of the Initial Sample and the Final Sample of Model 1

Table S5.1 displays average descriptive statistics for the defending networks comparing the full sample of 93 classes with the 44 classes for which model 1 reached convergence. Across all waves, the final sample of model 1 showed slightly larger values for the outdegree, the standard deviation of outdegree, and the standard deviation of indegree compared to the initial sample. Hamming distances were larger in the final sample, suggesting greater observed change over time. Levels of reciprocity, transitivity and same-gender nominations, as well as Jaccard indices, were similar across samples.

Table 2 presents descriptive statistics for victimization, popularity, and defending across the three waves for the full sample of 93 classes and the 44 classes for which model 1 converged. The proportion of victimized students among all participants was similar across the samples. The proportion of victims with at least one defender, as well as the proportion of students nominated as defender at least once and the number of victims defended by a defender was slightly larger in the final sample of model 1. Changes in victimization were overall similar between the samples, but the reduction in victimization among all victims was slightly larger in the full sample than in the final sample of model 1. Average levels of baseline popularity were comparable between the full sample and the final sample of model 1.

Table S5.1

Descriptive statistics of defending networks per wave for the initial sample and the final sample of model 1

	Wave 1 *M* (*SD*)		Wave 2 *M* (*SD*)		Wave 3 *M* (*SD*)	
	N = 93	N = 44	N = 93	N = 44	N = 93	N = 44
Outdegree	0.63 (0.46)	0.70 (0.45)	0.50 (0.32)	0.53 (0.31)	0.40 (0.34)	0.48 (0.37)
SD Outdegree	1.43 (0.94)	1.62 (1.00)	1.22 (0.78)	1.29 (0.74)	1.07 (0.89)	1.26 (0.98)
SD Indegree	0.65 (0.28)	0.70 (0.31)	0.62 (0.25)	0.67 (0.28)	0.52 (0.26)	0.57 (0.27)
Reciprocity	.35 (.36)	.35 (.35)	.34 (.38)	.34 (.39)	.15 (.30)	.14 (.27)
Transitivity	.41 (.30)	.42 (.27)	.39 (.36)	.42 (.37)	.39 (.40)	.39 (.40)
Same-gender nominations	.86 (.22)	.90 (.15)	.81 (.26)	.83 (.24)	.81 (.24)	.82 (.22)

	Wave 1→2 *M* (*SD*)		Wave 2→3 *M* (*SD*)	
	N = 93	N = 44	N = 93	N = 44
Hamming distance	7.73 (6.97)	9.82 (8.00)	6.31 (6.46)	8.07 (7.37)
Jaccard index	.32 (.27)	.31 (.21)	.29 (.28)	.30 (.29)

Note. N = 93 classes: initial sample for which social network analysis was computed; N = 44 classes: final sample of model 1.

Table S5.2

Descriptive statistics of victimization, popularity defending per wave for the initial sample and the final sample of model 1

	Wave 1		Wave 2		Wave 3	
	N = 93	N = 44	N = 93	N = 44	N = 93	N = 44
Number of participants with victimization data	1342	682	1337	693	1308	666
Victimization across all participants *M* (*SD*)	0.56 (0.95)	0.56 (0.96)	0.59 (1.02)	0.62 (1.08)	0.51 (1.00)	0.54 (1.03)
Proportion of victimized students among all participants	.34	.34	.35	.35	.29	.30
Proportion of victims with at least one defender	.78	.80	.71	.73	.69	.72
Proportion of students nominated as defender at least once	.47	.52	.39	.40	.33	.38
Number of victims defended by a defender *M* (*SD*)	1.42 (0.68)	1.44 (0.72)	1.33 (0.60)	1.37 (0.68)	1.25 (0.56)	1.29 (0.61)

	Wave 1→2		Wave 2→3	
	N = 93	N = 44	N = 93	N = 44
Change in victimization between waves across all participants *M* (*SD*)	0.05 (0.95)	0.05 (0.94)	-0.07 (0.97)	-0.05 (0.98)
Change in victimization among all victims *M* (*SD*)	-0.35 (1.26)	-0.29 (1.27)	-0.54 (1.26)	-0.50 (1.33)
Change in victimization among victims with at least one defender *M* (*SD*)	-0.32 (1.28)	-0.20 (1.30)	-0.57 (1.25)	-0.48 (1.35)
Change in victimization among victims without defenders *M* (*SD*)	-0.44 (1.17)	-0.62 (1.10)	-0.48 (1.30)	-0.55 (1.30)
Baseline popularity (proportion) among all participants *M* (*SD*)	0.15 (0.17)	0.14 (0.16)	0.14 (0.16)	0.12 (0.14)
Baseline popularity (proportion) among all defenders *M* (*SD*)	0.16 (0.17)	0.16 (0.16)	0.17 (0.17)	0.15 (0.16)

Baseline popularity (proportion) among all victims *M* (*SD*)	0.14 (0.16)	0.14 (0.15)	0.14 (0.16)	0.13 (0.15)

Note. N = 93 classes: initial sample for which social network analysis was computed; N = 44 classes: final sample of model 1.

S6: Additional Analyses Model 2a: Coding Defender Status as a Victim Attribute

Because only a limited number of classes were available for estimating the interaction between *defending outdegree → victimization* and the baseline popularity of the defenders, we conducted an additional analysis in which defender status was modeled as a victim-level attribute. Specifically, we created a time-varying covariate that, for each student at T1 and T2, represented the total popularity of all nominated defenders. For each time point, we summed the popularity scores of all individuals who were nominated as defenders by that student. The covariate was coded as NA in cases where no defenders were nominated (i.e., when the corresponding row in the defending network matrix was coded entirely as 0, 10, or NA). This time-varying covariate was added to the RSiena data object, and we included an interaction term between *defending outdegree → victimization* and the *effFrom* effect of the covariate.

The model converged in 31 classes, of which 27 were retained for the estimation of the interaction effect – five more than in Model 2a. As shown in Table S5.1, the results were consistent with those of Model 2a: the interaction effect was not statistically significant.

Table S6.1

Results of the meta-analysis across classes for model 2a with defender status as a victim attribute

	N	Est.	*SE*
Structural & covariate network effects			
Outdegree (density)	25	-2.58	0.22***
Outdegree Activity	28	0.54	0.08***
Same sex	30	0.33	0.12**
Behavior effects			
Linear	31	-0.97	0.10***
Quadratic	30	0.26	0.06***
Network → behavior			
D indegree → V	30	0.07	0.07
D indegree → V x baseline popularity defender	27	0.03	0.09

Note. Fixed rate of change effects were omitted from the table. *N* = number of classes included in the meta-analysis for this parameter. ** $p < .01$; *** $p < .001$ (two-tailed tests).

S7: Grade Effects

Table S7.1

Grade effects on the parameters of interest

	N	*b*	*se*	*z*	CI *LL*	CI *UL*
Model 1						
D outdegree → V	38					
intercept		0.12	0.21	0.58	-0.28	0.52
grade		-0.01	0.04	-0.38	-0.08	0.06
Model 2a						
D outdegree x baseline popularity defender → V	22					
intercept		-0.47	1.07	-0.44	-2.57	1.63
grade		0.08	0.18	0.44	-0.27	0.42
Model 2b						
D outdegree x baseline popularity victim → V	28					
intercept		-0.31	1.90	-0.16	-4.03	3.42
grade		0.01	0.35	0.02	-0.68	0.69
Model 2c						
D outdegree x baseline popularity defender → V	15					
intercept		-0.89	1.81	-0.49	-4.44	2.67
grade		0.14	0.30	0.47	-0.45	0.74
D outdegree x baseline popularity victim → V	15					
intercept		-2.19	2.66	-0.83	-7.40	3.01
grade		0.32	0.49	0.65	-0.64	1.28

Note. D = Defending (network). V = Victimization (behavior). *N* = number of classes included in the meta-analysis for this parameter. None of the effects were statistically significant; significance threshold: $p < .05$ (two-tailed).

References

Huitsing, G., Snijders, T. A. B., Van Duijn, M. A. J., & Veenstra, R. (2014). Victims, bullies, and their defenders: A longitudinal study of the coevolution of positive and negative networks. *Development and Psychopathology*, *26*(3), 645–659. https://doi.org/10.1017/S0954579414000297

Ripley, R. M., Snijders, T. A. B., Boda, Z., Vörös, A., & Preciado, P. (2024). Manual for RSiena. *University of Oxford. Department of Statistics.* https://www.stats.ox.ac.uk/~snijders/siena/RSiena_Manual.pdf
